# Service Need Utilization and Unmet Service Needs of Grandmothers Living With or Raising Grandchildren

**DOI:** 10.1093/geroni/igaa057.2057

**Published:** 2020-12-16

**Authors:** Carol Musil, McKenzie Wallace, Alexandra Jeanblanc

**Affiliations:** Case Western Reserve University, Cleveland, Ohio, United States

## Abstract

This study explores the service need utilization and unmet service needs of a nationwide sample of 284 grandmothers living with/raising grandchildren, and the relationships with service use/need, perceived stress, reward, and appraisals of their current living environment for themselves and their grandchildren. Participants were asked whether they currently used, had unmet need for, or did not need 25 different support services, including babysitting, financial assistance, legal assistance, family therapy/communication, among others. Overall, 89.5% (N=255) were receiving at least 1 service (mean = 3.4, range 0-18), and 89.1% (N=253) reported having at least 1 unmet service need (7.4, range 0-23). Receiving services was positively correlated with psychosocial resources, but not with appraisals of stress, reward, or living situation. Unmet service needs were inversely correlated with psychosocial resources, reward, and appraisals of living situation. Implications of these varying patterns will be discussed. Part of a symposium sponsored by the Grandparents as Caregivers Interest Group.

